# Scientific Items

**Published:** 1887-08

**Authors:** 


### SCIENTIFIC ITEMS.

**Hypnotism.**—A series of the most extraordinary experiments in hypnotism, made, under the direction of Dr. Charcot, by his assistant, Dr. Babinski, of the Salpetriere Hospital, surpass all hitherto conceived possibilities in medical science and cause a profound sensation in Parisian society. These experiments prove as perfectly practicable the transmission by magnetism from one person to another of ceitain nervous phenomena, such as dumbness, paralysis of the legs and arms, violent pains and coxalgia, and the final elimination of the evil from the original sufferer. These cures seem at first sight to be nothing short of miracles. As many fantastic and more or less exaggerated accounts of these experiments have appeared in the Parisian papers, I resolved to go at once to the fountain head authority, and called upon the famous Dr. Charcot himself at his magnificent mansion, on the Boulevard Saint-Germain.—*American Analyst.*

**An Incandescent Gas-Lamp.**—A novel lamp, which seems to combine in a measure the advantages of both gas and electricity, was invented some months ago by Dr. Auer von Welsbach, of Vienna. Its construction is very simple, consisting of a common Bunsen gas-lamp, in which is burnt a mixture of gas and air. This produces a very hot non-luminous flame ; but, by introducing into it a sort of network of mineral matter, it become incandescent, giving a bluish-white light, which is perfectly steady, and free from smoke.

The composition of this incandescent network is in part a secret; but the inventor claims that it is prepared by soaking a piece of coarsely woven cloth in a solution of the salts of zirconium, lanthanum, and yttrium. When this cloth is exposed to the flame, all the organic matter is consumed, leaving the oxides of the above-mentioned metals, which preserve the original form of the cloth. These, when heated, shine with a brilliant light, similar to the familiar calcium light, though less powerful.

The network tubes—or hoods, as the inventor calls them, only cost about a cent apiece, and will last for a thousand hours. It is claimed that the amount of gas burned is diminished one-half, with the same light-giving power; but practical trials will be needed to determine this. If the composition of the incandescent "hoods" has been given correctly by the inventor, an important practical use will have been found for some rather rare metals, which have hitherto been only of theoretical interest.—*Popular Science News.*

**Exhibition of Lighting-Apparatus.**—At a lecture recently delivered before the students of Boston University, there were exhibited, according to the press reports, the Persian pastilles of saturated sandstone, looking like cigars in candlesticks; the hollow soapstone filled with lard and wick, to give light in arctic lands, where half a year is wrapped in dusk; the tiny pyx lamps, worn in the belts of monks during their midnight vigils, centuries ago; the skulls of animals, which formed the designs for lamps in ruder ages; the gorgeous ecclesiastical candlestick; Florentine blendings of art and utility; quaint-looking lamps, that served the fathers and mothers of New England in colonial days; and the whale-oil and naptha lamps, beside which the good wives for generations did the family needlework and spinning. There, also, stood that modest object, the sperm candle, which is still looked up to as the unit of estimate in photometric work; the wax candle, which holds yet a high place on the altar and in the home; and all the modern devices of the arc and incandescent appliances of electricity which have come to us like a luminous progeny from the brains of Davy and Faraday.

"If you were to ask me," said the lecturer, as he touched a tiny earthen lamp with a rude wick hanging from it; "if you were to ask me who lit this lamp last, I think I could hardly tell you, because it was last used in the buried city of Pompeii, two thousand years ago.—*Popular Science News.*

**A Gas Locomotive.**—In Melbourne, Victoria, says the *Journal of Commerce and Intercolonial Trade*, a gas locomotive has been running for several months on one of the tramways, so far to the satisfaction of all concerned. The coal gas is carried in four copper containers, about six feet long by sixteen inches in diameter, which, as the gas is compressed to about fifteen atmospheres, hold two hundred and eighty cubic feet, or sufficient for a run of fifteen miles. In practice, the gas has rarely been pressed to more than a hundred pounds, as that gives an ample supply to run the locomotive and its car twice on its journey. The reservoirs or containers are refilled as required at the station; and the average consumption of gas per day of about eight trips, or forty miles, is seven hundred and two cubic feet, which in London would cost about forty-four cents. The locomotive weighs four and one-half tons, and the car thirty-five hundred weight, an Otto gas engine being the motor.—*Popular Science News.*

**Crystals of Gold.**—The *Microscope* says: "Although crystals occur in nature, they have never been produced artificially. By allowing a solution of the double chloride of gold and sodium to stand, Mr. W. N. Allen has observed that perfect little regular three and six-sided tablets of metallic gold are slowly deposited. The crystals were about 0.003 of an inch in diameter, and the upper surface shows a strong reflection."—*National Druggist.*

**Magnet.**—It is said in one of the French schools there is a natural magnet which is capable of lifting four times its own weight. This is a pretty *heavy* story.

A writer in *Scientific American* asks how to make an enamel on steel, one that will dry quick and black. A. Use Japan varnish and bake in an oven at 250° temperature.

